# Exercise and associated features with low-level exercise among doctors

**DOI:** 10.1016/j.clinsp.2023.100282

**Published:** 2023-10-03

**Authors:** Siriwan Tangjitgamol, Paisan Bunsiricomchai, Watcharagan Kaewwanna, Natapon Ativanichayapong, Sumonmal Manusirivithaya

**Affiliations:** a Research Center, MedPark Hospital, Bangkok, Thailand; b Cardiology, Center, MedPark Hospital, Bangkok, Thailand

**Keywords:** Dietary fiber, Exercise, Health surveillance, Physicians

## Abstract

**Background:**

Physical activity had been reported among doctors-in-training, with very few focusing on attending doctors. This study aimed to evaluate the physical activity, particularly exercise, of Thai doctors and features associated with low-level exercise.

**Materials and methods:**

Thai doctors who participated in the hospital's CSR service, which was conducted between Feb 14, 2022, to Oct 31, 2022, were verbally informed along with an information sheet about this parallel study. The doctors who consented to participate provided their personal data on diets, work hours, illnesses, health surveillance, stress, state of well-being, and exercise. To investigate the association with clinical features, the exercise level was categorized as low-level (≤ 2 days/week) or high-level (≥ 3 days/week).

**Results:**

The median age of 1187 doctors was 45.0 years. Slightly over half were female (55.4%) or worked ≤ 40 hours/week (55.3%). The presence of illnesses was found in 55.9%, irregular health surveillance in 29.0%, no/low fiber diet in 19.2%, overweight/obesity in 28.0%, and moderate/severe stress in 25.9%. Among the doctors who provided exercise data, 29.8% had a high-level of exercise and 49.0% had a low-level. Independent features associated with low-level exercise were no/low fiber diet (aOR = 3.01), irregular health surveillance (aOR = 2.41), age ≤ 60 years (aOR = 1.82), female gender (aOR = 1.79), and work > 40 hours/week (aOR =  1.76).

**Conclusions:**

Only 30% of Thai doctors had a high level of exercise. The significant independent factors for low-level exercise were younger age, female, working load, irregular health surveillance, and no/ low fiber diet.

## Highlights

• Exercise, one of the most crucial and beneficial health habits, is not a common practice among the general population.
• Data on exercise were mainly from trainees, with few from professional doctors.
• Nearly half of the 1187 doctors in the study had low-level exercise with young age, female, hard work, poor diet, and irregular health surveillance as risk factors.

## Introduction

The World Health Organization (WHO) has recommended daily physical activity for everyone of any age.^1^ Health benefits from physical activity are well recognized that the activity can reduce the risks of several Non-Communicable Diseases (NCD), including cardiovascular disease, hypertension, cancers, diabetes mellitus, prevention of falls, and reduction of anxiety and depression/stress.^1^ Despite a large body of evidence about the benefits of physical activity, more than a quarter of the world's adult population was insufficiently active.^1^ Physical inactivity was responsible for more than 10% of NCDs and 9% of premature death.^2^

Health promotion and prevention of illnesses are the frontline services of medical personnel, including doctors, nurses, and other public health practitioners. Health promotion includes, but is not limited to a healthy diet, adequate physical activity, exercise, and maintaining a good mental state or well-being. A doctor who generally serves as a team leader certainly positively impacts the behavior of other team members and their patients. Many studies revealed that doctor's physical activity habit influenced their counseling practice to their patients regarding physical activity.^3^, ^4^, ^5^ The healthcare providers who were physically active or had positive attitudes towards physical activity were more likely to counsel better and promote physical activity to their patients compared to those who were not active themselves.

An individual's physical activity depends on many factors, such as his or her basic health and attitude as well as the lifestyles of the family members, peers, associates at work, availability, type of work and responsibility, residence, and social environment, etc. Focusing on availability and responsibility, a doctor has specific tasks that are different from other medical personnel. Compared to the number of populations, the adequacy of doctors is a significant issue in determining their availability. The WHO has reported that the ratio of doctors per 1000 population was 3.53 globally.^6^ Conversely, the National Statistical Office of Thailand reported an unproportionable ratio of one doctor per 1680 population.^7^ Undoubtedly, this would impact not only the population's health but the health of the doctors as well.

Most studies in other countries and few in Thailand reported various levels or types of physical activities among the doctor-in-training (medical students or residents), the barriers for not having or inadequate activities, and their impact on health.^8^, ^9^, ^10^, ^11^ The patterns of physical activity among professional doctors may vary due to differences in lifestyles, work schedules, and responsibilities compared to other medical professions and doctors in training. However, there have been no studies that specifically addressed the physical activity levels of professional doctors or explored the reasons behind their lack of exercise. This study aimed to assess the physical activity of Thai doctors, focusing on exercise frequency, and clinical features or risk factors associated with low-level exercise.

## Materials and methods

This study was conducted in parallel to the hospital's Corporate Social Responsibility (CSR) project Save Doctors’ Heart, which was conducted between February 14, 2022, to October 31, 2022. The research was approved by the Institutional Review Board (COA-MPIRB 003/2022).

The project participants were Thai doctors aged between 35‒75 years without any congenital heart diseases. In brief, the project's medical services comprised physical examination of weight, height, blood pressure, and cardiovascular system examination. Basic laboratory testing and selected cardiac testing were performed according to the risk features, abnormal basic testing, and at the discretion of the examining cardiologist. The details of clinical testing were presented in our main cardiovascular report.

The individuals who joined our CSR project were invited to the study. Data of the doctors, who had read an information sheet about the study and verbally consented to participate by voluntarily giving information, were collected from electronic medical charts and a one-page questionnaire about personal, work and health habits, which were self-answered upon entering the study. Only the doctors who had answers to physical activity (exercise in particular) were included in this study.

Data collected included age, gender, marital status, monthly family income, number of workplace, primary (major) workplace, weekly work hours, which was summed from total working hours per week, health coverage, history of health checkup, health habits of alcohol & caffeine consumption, the relative quantity of fiber diet (minimal, low, moderate, high), smoking, exercise (open-end question), stress (none, mild, moderate, severe), history of illness, and personal characteristics for well-being (generosity, positive outlook to life, attention or mindfulness for problem-solving, and resilient to life difficulty; each was scored one as least to 4 as most). The Body Mass Index (BMI) was also collected.

Each characteristic feature was grouped as the following: monthly family income as more or less than 200,000 Baht (5730 USD); primary workplace as private or public; weekly work hours as ≤ 40 hours or more; history of health checkup as regular annually or irregular (when there were symptoms or at convenience); dietary fiber consumption as good (moderate/high) or fair (minimal/low); stress as none/mild or moderate/severe. The frequency of exercise was categorized as none, 1‒2 days/week, and ≥ 3 days per week and grouped as low-level (≤ 2 days/week) or high-level (≥ 3 days/week). The well-being scores were summed up and grouped as good (higher score than median) or fair. The BMI was categorized as underweight (< 19 kg/m^2^), normal (19‒26 kg/m^2^), overweight (> 26‒30 kg/m^2^), or obese (> 30 kg/m^2^) before grouping into non-obese or obese.

Statistical analyses were performed using IBM SPSS Statistics 22.0 was used (IBM Corp., Armonk, NY, USA). Continuous variables were presented as mean ± Standard Deviation (SD) for normally distributed data or as median and ranges. Categorical variables were presented as frequency and percentage. A univariate analysis, by Chi-Square or Fisher exact test as appropriate, was performed to identify clinical features, including BMI, which may be associated with exercise. Significant factors found from univariate analysis were included in the multivariate logistic regression analysis to explore independent factors associated with exercise. Data on risk factors of low-level exercise were presented as Odds Ratio (OR) with a 95% Confidence Interval (95% CI). A *p*< 0.05 was considered statistically significant.

## Results

Slightly over 1500 doctors had registered for the Save Doctors’ Heart project. Due to many reasons, as detailed in another report that would be presented elsewhere (Work and health habits of Thai doctors), 1244 who had read the information sheet about the parallel research work agreed to participate in the study. However, only 1187 provided data on exercise and were included in this study.

Data on age and gender were available from all 1187 doctors. The median age was 45.0 years (range 35‒73 years). Slightly more than half (52.4%) were between 35‒45 years old, whereas nearly one-third (31.6%) were between 46‒59 years, and the remaining (16.0%) were 60 years or over. Slightly more than half were female (55.4%).

The responses to the questions of personal and work habits varied. The personal and working data of the participating doctors are shown in Table 1. Approximately two-thirds (63.3%) were or had been married, and more than half (57.4%) had an average monthly family income of more than 200,000 Baht (5730 USD). The majority (85.5%) had only one primary (main) workplace, with approximately one-third being either in private or public hospitals (36.9% and 34.5%, respectively). The authors summed up the working hours of each participant each week. The median weekly work hours was 40.0 (range 1 to 168 hours), with less than half (44.7%) working > 40 hours per week.

**Table 1:** Basic characteristics of participants.

Characteristic features	n	%
**Personal**		
Age in years (*n*= 1187)		
35‒45	622	52.4
46‒59	375	31.6
≥ 60	190	16.0
Gender (*n*= 1187)		
Female	658	55.4
Male	529	44.6
Marital status (*n*= 709)		
Single	230	32.4
Married	449	63.3
Separate/divorced	30	4.3
Family income, Baht (approximate USD^a^) (*n*= 666)		
< 100,000 (< 2860)	198	29.7
100,000‒200,000 (2860‒5730)	86	12.9
> 200,000 (> 5730)	382	57.4
**Work habit**		
Number of workplaces (*n*= 626)		
1 place	535	85.5
2 places	61	9.7
> 2 places	30	4.8
Primary workplace (*n*= 626)		
Private hospital	231	36.9
Public hospital	216	34.5
Private clinic	129	20.6
Public health unit	22	3.5
Others, not otherwise specified	28	4.5
Work hours per week (*n*= 685)		
≤ 40	379	55.3
> 40	306	44.7

a 34.9281 Baht = 1 USD. https://www.bot.or.th/thai/_layouts/application/exchangerate/exchangerate.aspx.

Apart from the history of illness to which all 1187 participants responded, other questions were reported at different rates. The number and percentages of the doctors’ health habits, health history, and health findings are shown in Table 2. No or low fiber diet was reported in 19.2%, alcohol or caffeine consumption in 10.7% and 68.6%, respectively, and almost all denied smoking habit. Focusing on exercise, 252 doctors (21.2%) reported no exercise. Among the other 935 doctors (78.8%) who reported that they have some exercises, the frequency was 1‒2 days/week in 582 (49.0%) and ≥ 3 days/week in 353 (29.8%). In total, 834 (70.2%) were grouped as no or low-level exercise (≤ 2 days/week) and 353 (29.7%) as high-level (≥ 3 days/week).

**Table 2:** Health habits, health history and health findings of the doctors.

Feature	n	%
**Health habits**		
Fiber diet (*n*= 705)		
Minimal	4	0.6
Low	131	18.6
Moderate	444	63.0
High	126	17.8
Alcohol (*n*= 1181)		
No	1055	89.3
Yes	126	10.7
Caffeine consumption (*n*= 1040)		
No	327	31.4
Yes	713	68.6
Smoking (*n*= 1186)		
No	1177	99.2
Smoked or ever smoked	9	0.8
Exercise (n =1187)		
None	252	21.2
1‒2 days/ week	582	49.0
≥ 3 days/ week	353	29.8
**Health history**		
History of health surveillance (*n*= 703)		
Regular	499	71.0
Irregular	204	29.0
History of illness (*n*= 1187)		
No	523	44.1
Yes	664	55.9
**Health findings**		
Stress (*n*= 1173)		
No	210	17.8
Mild	660	56.3
Moderate	288	24.6
Severe	15	1.3
Sate of well-being^a^ (*n*= 705)		
Fair (score < 13)	330	46.8
Good (score ≥ 13)	375	53.2
Body mass index (kg/m^2^)		
<24.9	849	72.0
25–29	275	23.3
≥ 30	55	4.7

a The median score of well-being was 13.0 (range 6‒16).

For health history and health findings, 71.0% reported regular health surveillance, and 44.1% did not have a history of any illnesses. Some degree of stress was reported in 82.2%: mild in 56.3%, moderate in 24.6%, and severe in only 1.3%. The median well-being score was 13, slightly over half categorized as good and 46.8% as fair. Slightly over one-fourth of the doctors in this study were overweight or obese (28.0%), whereas the remaining were normal or underweight.

The authors investigated the association of characteristic features of the doctors, their health habits, and health findings with the level of exercise (Table 3). By univariate analysis, low-level of the exercise was found significantly more frequent among the doctors with some features compared to their comparative groups (Odd Ratio; OR): age ≤ 60 years (73.6% vs. 51.9%; OR=2.58, *p*-value < 0.001), female gender (73.9% vs. 65.8%; OR = 1.47, *p*-value = 0.002), overweight/obesity (75.5% vs. 69.9%; OR = 1.43, *p*-value = 0.014), > 40 weekly work hours (84.6% vs. 74.7%; OR = 1.90, *p*-value=0.001), irregular health surveillance (87.7% vs. 74.9%; OR = 2.39, *p*-value < 0.001), only fair fiber diet (91.1% vs. 75.8%; OR = 3.27, *p*-value < 0.001), and moderate/severe stress (77.2% vs. 68.0%; OR = 1.59, *p*-value = 0.003). Of note, the doctors without any history of illnesses tended to do less exercise. The authors additionally explored the exercise and features of age and weekly work hours in combination. Low-level exercise was associated with doctors younger than 60 compared to older people, regardless of their work hours. Among those who worked ≤ 40 hours/week, low-level exercise was found in 77.1% (*n* = 236/306) of the younger age group vs. 63.0% (*n =*= 49/73) of the older age group (*p =*= 0.013). The corresponding figures among those who worked > 40 hours/week were 86.0% (*n =*= 245/285) vs. 66.7% (*n =*= 14/21) (*p =*= 0.018).

**Table 3:** Exercise level according to clinical features by uni- and multi-variate analyses.

Feature	n	Exercise		Unadjusted OR (95% CI)	p	Adjusted OR (95% CI)	*p*-value
		High level, *n* (%)	Low level, *n* (%)				
**Personal data**							
Age, year (*n*= 1187)							
> 60	181	87 (48.1)	94 (51.9)	1	< 0.001	1	0.019
35‒60	1006	266 (26.4)	740 (73.6)	2.58 (1.86‒3.56)		1.82 (1.102‒3.01)	
Gender (*n*= 1187)							
Male	529	181 (34.2)	348 (65.8)	1	0.002	1	0.005
Female	658	172 (26.1)	486 (73.9)	1.47 (1.15‒1.89)		1.79 (1.194‒2.70)	
Body mass index (*n*= 1179) (kg/m^2^)							
Under- to normal	849	270 (31.8)	579 (69.9)	1	0.014	1	0.106
Overweight/ obese	330	81 (24.5)	249 (75.5)	1.43 (1.07‒1.92)		1.45 (0.92‒2.27)	
Marital status (*n*= 709)							
Single/Divorced	260	56 (21.5)	204 (78.5)	1	0.961	‒	‒
Married/Separate	449	96 (21.4)	353 (78.6)	1.009 (0.70‒1.46)		‒	‒
Monthly Family income, Baht (approximate USD^a^) (*n*= 666)							
≤ 200,000 (< 2860)	284	67 (23.6)	217 (76.4)	1	0.369	‒	‒
> 200,000 (> 5730)	382	79 (20.7)	303 (79.3)	1.18 (0.82‒1.71)		‒	‒
Work hours per week (*n*= 685)							
≤ 40	379	97 (25.6)	282 (74.7)	1	0.001	1	0.007
> 40	306	47 (15.4)	259 (84.6)	1.90 (1.29‒2.79)		1.76 (1.16‒2.65)	
Primary workplace* (*n*= 626)							
Public hospital	238	55 (23.1)	183 (76.9)	1	0.726	‒	‒
Private hospital	388	85 (21.9)	303 (78.1)	1.07 (0.73‒1.58)		‒	‒
No of workplace (*n*= 626)							
1	535	119 (22.2)	416 (77.8)	1	0.860	‒	‒
More than 1	91	21 (23.1)	70 (76.9)	1.05 (0.62‒1.78)		‒	‒
**Health data**							
Health surveillance (*n*= 703)							
Regular	499	125 (25.1)	374 (74.9)	1	< 0.001	1	< 0.001
Irregular	204	25 (12.3)	179 (87.7)	2.39 (1.50‒3.81)		2.41 (1.48‒3.94)	
History of illness							
Yes	664	206 (31.0)	458 (69.0)	1	0.275	‒	
None	523	147 (28.1)	376 (71.9)	1.15 (0.89‒1.48)		‒	
Fiber diet (*n*= 706)							
Good	571	138 (24.2)	433 (75.8)	1	< 0.001	1	< 0.001
Fair	135	12 (8.9)	123 (91.1)	3.27 (1.75‒6.09)		3.01 (1.58‒5.73)	
Stress (*n*= 1173)							
No to mild	870	278 (32.0)	592 (68.0)	1	0.003	1	0.053
Moderate/severe	303	69 (22.8)	234 (77.2)	1.59 (1.18‒2.16)		1.61 (1.99‒2.61)	
State of well-being^b^ (*n*= 705)							
Good	375	86 (22.9)	289 (77.1)	1	0.252	‒	‒
Fair	330	64 (19.4)	266 (80.6)	0.81 (0.56–1.16)		‒	‒

a 34.9281 Baht = 1 USD. https://www.bot.or.th/thai/_layouts/application/exchangerate/exchangerate.aspx.

b The median score of well-being was 13.0 (range 6‒16).

By multivariate analysis, only five features remained significant. The risk of these features (adjusted Odds Ratio; aOR) for low-level exercise in descending orders were fair fiber diet (aOR = 3.01, *p*-value < 0.001), irregular health surveillance (aOR = 2.41, *p*-value < 0.001), age ≤ 60 years (aOR = 1.82, *p*-value = 0.019), female gender (aOR = 1.79, *p*-value = 0.005), and > 40 weekly workhours (aOR = 1.76, *p*-value = 0.007). Moderate to severe stress was only marginally significant (aOR=1.61, *p*-value = 0.053).

## Discussion

Although many studies have evaluated doctors’ physical activity, most assessed the impact of this activity on their counseling to the patients.^3^, ^4^, ^5^^,^^11^^,^^12^, ^13^, ^14^, ^15^ Only a few studies that focused on doctors’ physical activity reported a prevalence ranging from 62% to 74%.^5^^,^^14^^,^^16^^,^^17^ The present study found that 78.7% of the Thai doctors had physical activity. This was slightly higher than previous reports.

The rate of physical activity in each study may vary depending on many factors such as the number and characteristics of the study population, the indicator and definition used, the method of study including the questions in the questionnaires, etc. For example, this study included Thai doctors in any healthcare unit (hospital, clinic and either private or public). Other studies conducted a survey study focusing on the physical activity of the doctors in various groups (medical students, residents, or doctors), in private practice or public primary health service.,^3^^,^^9^^,^^13^^,^^15^, ^16^, ^17^, ^18^ various ethnic groups.^16^

The WHO has recommended that adults aged 18‒64 years should do a minimum of various durations:  150–300 minutes of moderate intensity, 75–150 minutes of vigorous-intensity, or an equivalent combination of them throughout the week.^1^ For adults ≥ 65 years, multicomponent physical activity three or more days a week is recommended.^1^ These benchmarks are, at times, difficult to genuinely obtain in a clinical study. Few authors assessed the physical activity by frequency (duration per day or days per week),^16^ whereas others were able to additionally assess the level of physical activity (moderate or strenuous).^5^^,^^12^, ^13^, ^14^^,^^17^ The present study used “exercise” to indicate physical activity. Data were collected from the hospital health assessment form, so the exercise was leveled by its frequency because the strenuousness or intensity was not available in detail.

The levels of exercise were categorized as either high- or low-level to assess their association with clinical features. The authors inferred that doctors who claimed to have no exercise should still engage in some physical activities through work and daily routines. Consequently, 21.3% of doctors who reported no exercise were combined with the 49% who exercised 1‒2 days per week, resulting in a total of 70% classified as “low-level” exercise. The remaining 30% were categorized as “high-level” exercise (> 3 days/week), which was notably lower compared to the 65% to 74% rates of moderate to vigorous or high-level physical exercise reported in other studies.^5^^,^^14^ The difference in findings could be attributed to the characteristics of the study population. Previous studies focused on primary care doctors. In contrast, the majority of our participants worked in hospitals, encompassing both private and public settings. Additionally, it is important to note that a direct comparison of the level of exercise or physical activity between the studies would be inappropriate due to the utilization of different assessment tools to measure the frequency and intensity of activity.^5^^,^^12^, ^13^, ^14^^,^^17^^,^^19^

Several other studies have examined and found an influence of a doctor's personal physical activity or attitudes and counseling practices concerning this topic with patients.^3^^,^^5^^,^^11^^,^^12^, ^13^, ^14^ For example, a large-scale study conducted in the US, involving nearly 2,000 doctors at different stages of their careers (566 attending doctors, 138 fellow doctors, 806 resident doctors, and 215 medical students), found that participants with normal BMI who engaged in vigorous physical activity felt more confident in counseling their patients on this issue.^13^ Of note, our study did not specifically focus on the impact of doctors' physical activity and their counseling practices. Instead, the authors aimed to explore various factors associated with low-level exercise among doctors. Upon evaluating multiple features, the authors found independent associated factors for low-level exercise included a fair fiber diet, irregular health surveillance, age ≤ 60 years, female gender, and working > 40 hours per week.

Few previous studies did not identify any association between physical activity and age, BMI, or smoking features.^12^^,^^19^ However, some of the significant associated factors with low-level exercise in our study were well recognized or had been reported in other previous studies. Different findings from each study might lie on characteristic features of the population, including gender distribution,^12^ region and cultures of the study,^13^ as well as the indicators and type of questionnaires used in each study.^5^^,^^12^^,^^13^^,^^16^^,^^17^^,^^19^

The doctors with long weekly work hours, which was associated with less exercise, were self-explanatory as these doctors may already be exhausted and had limited free time for physical activity. The poor health habits of a low-fiber diet and irregular health surveillance were also identified to have a higher chance of low-level exercise than other features. Some authors also reported that 25% of their doctors had irregular health checkups and found associated features of unhealthy food, beverages, and exercise habits.^20^

The authors also observed a higher prevalence of low-level exercise among female doctors. There were inconsistent data on the influence of gender on exercise. Several studies found that female medical students had less frequent physical activity than male counterparts.^14^, ^15^, ^16^^,^^18^^,^^21^ However, a study by Blake et al. from the UK, which included medical and nursing students, reported no different influence of gender (and discipline of doctor or nurse) on exercise.^15^

Stress and overweight or obesity may be viewed as a cause-and-effect or a bidirectional relationship. Exercise is generally well-recognized to elevate mood through the release of endorphins or a neurotransmitter inducing positive feelings.^22^ A direct association between a high level of exercise and less stress found in our study was consistent with previous studies.^10^^,^^11^ Doctors with moderate/severe stress may have other important matters to deal with, so not willing or available time for exercise.

Likewise, inadequate physical activity is associated with many non-communicable diseases, including overweight and obesity.^13^^,^^16^ In reverse, these illnesses, overweight or obesity, could be the reasons or barriers to inadequate exercise, as reported in previous studies. ^13^^,^^16^ Contrary to the previous statement, our study found that doctors with a history of illness tended to have high-level exercise more frequently than healthy individuals. This may be explained by the fact that these doctors who perceived themselves as being in poor health may be more attentive to improving their health through exercise.

The authors were surprised by one finding in our study that younger age was an independent risk factor for low-level exercise. Generally, younger individuals should be more physically active than the older group. No other influencing factors were identified, and the authors did not know the underlying reason for this finding. Instead, the authors postulated that younger doctors frequently have a busy life during their financial and social building-up period, so they have less time for exercise.

The authors were aware of some limitations in our survey study. Firstly, the authors used the frequency of exercise as the primary indicator, but not its strenuous or vigor. This might have led to an inaccurate comparison with other previous studies that focused on physical activity using a comprehensive questionnaire. Furthermore, certain personal factors such as health vices (alcohol or smoking) and personal information (monthly household income) were not entirely available. Aside from the missing information, the authors were unable to verify these personal data.

Nevertheless, this study had some strengths. Firstly, it was the first study conducted in Thailand that specifically examined physical activity among doctors. With over 1,000 participants, the sample size was considerable, making our data highly informative. Secondly, the study identified factors associated with low-level exercise. These unfavorable factors can be disseminated to relevant individuals or organizations, raising awareness and prompting necessary actions. Doctors with risk factors should remain vigilant and make lifestyle modifications accordingly. The importance of a fiber-rich diet, adopting a healthy work-life balance, and undergoing regular health surveillance should be emphasized.

## Conclusions

The majority of Thai doctors (79%) who took part in our study reported engaging in regular exercise. However, only 30% maintained a high level of exercise (more than 3 days per week). The key independent factors associated with low-level exercise included being 60 years of age or under, female, working more than 40 hours per week, irregular health check-ups, and having a diet low in fiber.

## Funding

This work was granted from MedPark Hospital Research Center Funds. The funders had no role in study design, data collection and analysis, decision to publish, or preparation of the manuscript.

## CRediT authorship contribution statement

**Siriwan Tangjitgamol:** Conceptualization, Supervision, Methodology, Funding acquisition, Formal analysis, Writing – original draft, Writing – review & editing. **Paisan Bunsiricomchai:** Conceptualization, Data curation, Writing – review & editing. **Watcharagan Kaewwanna:** Methodology, Data curation, Writing – original draft, Writing – review & editing. **Natapon Ativanichayapong:** Data curation, Formal analysis, Writing – review & editing. **Sumonmal Manusirivithaya:** Validation, Visualization, Writing – review & editing.

## Declaration of Competing Interest

The authors declare no conflicts of interest.
